# The Examining Board

**Published:** 1868-07

**Authors:** 


### THE EXAMINING BOARD.

The Board of Examiners appointed by the Ohio State Dental Society in pursuance of the law passed by the legislature of the State of Ohio, at its recent session, to regulate the practice of dentistry in this State, met at the Neil House, Columbus, on the 8th inst., at 10 o'clock, A. M., in answer to the call of the Chairman of the Committee.

There were present, Drs. M. DeCamp, F. H. Rehminkle, J. Taft, W. P. Horton and C. H. Harroun.

The object of the meeting was to organize the Board, and make such arrangements and regulations as may be required for carrying out the provisions of the law. The organization was effected by electing Dr. J. Taft, President, and Dr. W. P. Horton, Secretary and Treasurer.

The duty of the Board is to examine all applicants as to their qualification to practice Dental Surgery, and to grant certificates to all having the proper attainments.

The next meeting of the Board will be held in Columbus, on the first Tuesday of December next, for the examination of applicants. T.

